# Screening of Lactic Acid Bacteria and Yeasts from Sourdough as Starter Cultures for Reduced Allergenicity Wheat Products

**DOI:** 10.3390/foods9060751

**Published:** 2020-06-05

**Authors:** Wenhui Fu, Wentong Xue, Chenglong Liu, Yang Tian, Ke Zhang, Zibo Zhu

**Affiliations:** 1 College of Food Science and Nutritional Engineering, China Agricultural University, Beijing 100083, China; fwh@cau.edu.cn (W.F.); CLliu@cau.edu.cn (C.L.); YT@cau.edu.cn (Y.T.); KZ@cau.edu.cn (K.Z.); 2 College of Civil Engineering and Architecture, Henan University of Technology, Zhengzhou 450000, China; zzb229@haut.edu.cn

**Keywords:** lactic acid bacteria, yeast, proteolysis, fermentation ability, wheat allergy

## Abstract

Previous researchers have shown the potential of sourdough or related lactic acid bacteria in reducing wheat allergens. However, there are no mixed or single cultures for producing reduced allergenicity wheat products. In this study, twelve strains of lactic acid bacteria and yeast isolated from sourdough were evaluated for their ability to hydrolyze proteins and ferment dough. Strain *Pediococcus acidilacticiXZ31* showed higher proteolytic activity on both casein and wheat protein substrates, and had strong ability to reduce wheat protein allergenicity. The tested *Saccharomyces* and non*-Saccharomyces* showed limited proteolysis. Strains *Torulaspora delbrueckii JM1* and *Saccharomyces cerevisiae JM4* demonstrated a higher capacity to ferment dough compared to other yeasts. These strains may be applied as starters for the preparation of reduced allergenicity wheat products.

## 1. Introduction

Wheat is a very economically important cereal worldwide and has significant nutritional value. However, wheat is also one of the most frequent causes of food allergy [1]. The most common immune reactions to wheat ingestion such as wheat allergy, celiac disease, non-celiac wheat sensitivity (NCGS), and wheat–related inflammatory bowel diseases (IBD) have an increasing prevalence around the world [2]. Wheat allergens belong to the group of gluten (gliadins and glutenins) and non-gluten fractions (albumin and globulin). Gluten accounts for 80% of the total wheat protein and is associated with coeliac disease (CD). Screening studies show a high prevalence of CD (between 1/100–1/300) among adults in most countries [3]. Avoiding the uptake of allergenic proteins in wheat is essential for people with these diseases. Therefore, food processing technologies have been widely investigated to manufacture hypoallergenic foods.

Thermal processing is one of the most important techniques for the food industry. Heat based processing has been widely explored and used to modify allergenic proteins. However, it becomes less appealing as allergenic attenuation can only be achieved at extreme temperatures [4,5,6,7]. Researchers have found that fermentation by using sourdough or isolated fungi and bacteria can affect allergenicity while maintaining a higher organoleptic quality and nutritional profile of the dough [8,9]. The diversity of microorganisms in sourdough have been relatively well recognized. Lactic acid bacteria (LAB) and yeasts in sourdough have been widely isolated and identified by using culture-dependent and culture-independent methodologies, encouraging further studies on them [10,11]. Cell-envelope proteinase (CEP), oligopeptide transport systems, and peptidases play vital roles in the protein degrading process by lactic acid bacteria. Protein hydrolysate generated by CEP or endogenous protease is transferred into cells and further degraded by peptidases [12,13]. Previous studies have shown the presence of different pools of peptidases in various LAB strains. Some strains have enzymes such as PepI and PepX peptidases, which can degrade the cyclic structure of proline in wheat allergens [14]. Yeast activity is mainly contributable to CO_2_ production. The amount of CO_2_ produced during fermentation influences the viscoelastic properties of wheat dough and may enhance the thiol-disulfide exchange reaction, thereby affecting gluten depolymerization [15,16,17]. Although members of the genus *Saccharomyces* or non*-Saccharomyces* have been reported to have limited ability to secrete external hydrolases, they are able to release cytoplasmic proteases to the surrounding medium due to the cell lysis and death [18].

The aim of this study was to evaluate the proteolysis and CO_2_ production capacity of lactic acid bacteria and yeasts isolated from Chinese traditional sourdough. The potential yeasts and bacteria screened in sourdough may provide a new prospect for the preparation of reduced allergenicity wheat products.

## 2. Materials and Methods

### 2.1. Micro-Organisms and Growth Conditions

Twelve strains isolated from Chinese traditional sourdough were studied to evaluate their proteolytic and CO_2_ production activities. The following species were used: *Enterococcus faeciumAH1* (AH1), *Lactobacillus paracaseiAH2* (AH2), *Lactobacillus plantarumLN5* (LN5), *Pediococcus pentosaceusGD4* (GD4), *Pediococccus acidilacticiXZ31* (XZ31), *Lactobacillus plantarumSX1* (SX1), *Lactobacillus sakeiGS6* (GS6), *Torulaspora delbrueckiiJM1* (JM1), *Pichia anomalaJM2* (JM2), *Issatchenkia orientalisJM3* (JM3), *Saccharomyces cerevisiaeJM4* (JM4), and *Saccharomyces cerevisiaeJM5* (JM5). De Man–Rogosa–Sharpe (MRS) broth medium (400 mL/500 mL culture bottles) was seeded with 2 mL of a one-day culture of LAB and incubated at 37 °C. Yeast inoculation amount was 4% (*v*/*v*) and grown in Yeast Peptone Dextrose (YPD) medium (200 mL/500 mL culture bottles) at 28 °C. All strains were incubated for 20 h to the stationary phase of growth.

### 2.2. Proteinase and Peptidase Activities

Twenty-hour-old cells in 400 mL of culture medium were harvested by centrifugation (6000 × g for 10 min at 4 °C) and washed twice in 20 mM Na-phosphate buffer (pH 7.0). The supernatant was lyophilized and stored at −80 °C for the determination of extracellular enzyme activity. According to a slightly modified existing procedure [19], the cells were sonicated in 50 mM Tris-HCl buffer (pH 7.5) containing 0.1% (*w*/*v*) lysozyme and 24% (*w*/*v*) sucrose to collect the intracellular extracts. Cellular fractions were freeze-dried and stored at −80 °C until use.

Lyophilized samples were dissolved (1:2.5 *w*/*v*) in 0.05 M Tris-HCl buffer (pH 7.5) and 0.05 M potassium phosphate buffer (pH 7.0) for proteinase and peptidase activities analysis, respectively [20,21,22]. Protein concentration was determined by the Bradford method [21]. Proteinase activity was determined as described by Hittu and Vasu [20] using casein (Solarbio Science & Technology Co. Ltd., Beijing, China) as the substrate. The reaction mixture contained 1 mL of substrate (1% *w*/*v*) dissolved in Tris-HCl buffer, 1 mL of the diluted samples, and incubated at 37 °C for 20 min. The reaction was then stopped by adding 2 mL of 0.4 M trichloroacetic acid. After centrifugation (5000 × g for 10 min at 4 °C), the supernatant was reacted with the Folin reagent, and the absorbance was measured at 680 nm. The results were compared with the standard curves obtained by using tyrosine. General aminopeptidase type N (EC 3.4.11.11; PepN), proline iminopeptidase (EC 3.4.11.9; PepI), and X-prolyldipeptidyl aminopeptidase (EC 3.4.14.5; PepX) activities were measured according to a previous study [22] using Leu-*p*-nitroanilidies, Pro-*p*-nitroanilidies, and Gly-Pro-*p*-nitroanilidies (Solarbio Science & Technology) as the substrate, respectively. The assay mixture contained 900 μL of 2.0 mM substrate in 0.05 M potassium phosphate buffer (pH 7.0) and 100 μL of diluted samples. After incubation at 30 °C for 1 h, the absorbance was measured at 410 nm. The data obtained were compared to standard curves set up by using *p*-nitroanilide. One unit of enzymatic activity was determined as the amount of enzyme required to release 1 μg of tyrosine or 1 μmol of *p*-nitroanilidies (NA) per min under the assay conditions.

### 2.3. Hydrolysis of Wheat Protein Extracts

Cells of LAB were harvested by centrifugation at 600 × *g* for 10 min at 4 ${}^{{}^{\circ}}C$, washed twice, and resuspended in 20 mM Na-phosphate buffer (pH 7.0) before use (optical density at 600 nm, ca. 2.5). Wheat protein hydrolysate was produced according to Raffaella et al. [14]. Briefly, 30 g wheat flour was suspended in 100 mL phosphate buffer (20 mM, pH 7.2–7.4). After 30 min of sonication on ice, the suspension was centrifuged (10,000× *g* for 10 min at 4 °C and sterilized by filtration (0.22-μm-pore-size Millex-GV; Millipore, Ireland). The assay mixture, containing 3 mL of wheat protein hydrolysate (1.25 mg/mL of proteins) and an equivalent volume of the cellular suspension (OD 600 = 2.5, ca. 10^9^ CFU/mL) was incubated at 37 °C for 0, 4, 8, 12, and 24 h under stirring conditions (100 rpm). The supernatant was recovered by centrifugation and used for sodium dodecyl sulfate-polyacrylamide gel electrophoresis (SDS-PAGE) and enzyme linked immunosorbent assay (ELISA). For SDS-PAGE analysis, the supernatant was mixed with loading buffer (5 × loading buffer, Solarbio Science & Technology Co, Ltd., Beijing, China). As described by Rao et al. [23], 15 μg proteins were loaded onto 5/12% polyacrylamide gel stained with Coomassie brilliant blue (R-250). After being destained in washing buffer (50% *v*/*v* methanol, 10% *v*/*v* acetic acid) overnight, the gray values were calculated using ImageJ software. The degree of protein hydrolysis was also estimated by absorbance according to Oliveira et al. [24]. The supernatant obtained in each time interval was mixed with equal volume of trichloroacetic acid (20%) and centrifuged 10,000× *g* for 10 min at 10 °C The results were expressed directly as absorbance values at 280 nm.

### 2.4. Immunological Analysis

The ELISA assay protocol was performed as described previously to assess the effect of LAB on the IgG-binding capabilities of wheat protein hydrolysate [25]. The purified wheat protein was used to immunize rabbits to obtain antiserum. The specific method of preparing the rabbits antisera can be seen in the Supplementary Materials. All of the experiments were approved by the animal ethics committee at China Agricultural University. The microplate was coated with 100 μL of protein hydrolysate (5 μg/mL) and incubated overnight at 4 °C. On the second day, wells were blocked with bovine serum albumin (1:100 *w*/*v* diluted in 0.02M Tris-buffered saline) and then added to rabbit serum (1:10000 *v*/*v* diluted in blocking buffer). After incubation with HRP-conjugated goat anti-rabbit IgG (1:500 *v*/*v* diluted in blocking buffer), the assay were performed by using a tetramethylbenzidine substrate kit (Beyotime Biotechnology, Shanghai, China). Subsequently, the reactions were measured with an absorbance at 450 nm. The absorbance of the sample at 0 h and 24 h is represented by X_0_ and X, and the IgG binding reduction is expressed as (X_0_ − X) × 100/X_0_.

### 2.5. Determination of CO_2_ Production

The CO_2_ release of the dough prepared with different yeasts was assessed by using the rheofermentometer F4 (Chopin, Villeneuve-La-Garenne Cedex, France). Commercial wheat flour (150 g), distilled water (50 g), and 25 mL yeast suspension (final concentration in the dough was ~10^7^ CFU g^−1^ dough) were mixed in a mixing machine (Joyoung JYS-N6, Jinan, China). Subsequently, the dough was placed immediately in the fermentation jars with a piston and 2000 g weight on it. The test was conducted at 30 ${}^{{}^{\circ}}C$ for 1 h. Each group was carried out in triplicate in dependent experiments.

### 2.6. Statistical Analysis

All data were processed using Origin 8.5 software. All experiments were carried out at least three times.

## 3. Results

### 3.1. Proteinase and Peptidase Activities

**Figure 1:**
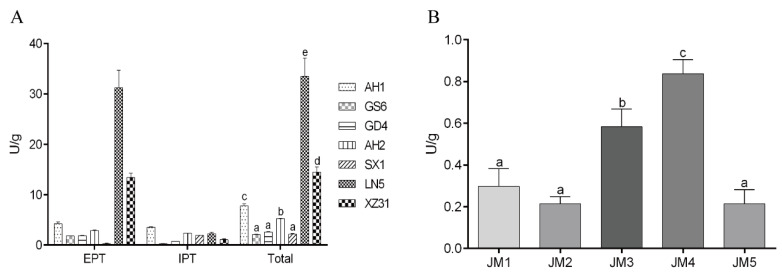
(**A**) The activities of extracellular proteinases (EPT) and intracellular proteinases (IPT) of seven lactic acid bacteria strains on the casein substrate. (**B**) The activities of intracellular proteinases of five yeasts on the casein substrate. Bars that do not share a common lowercase letter differ significantly (*p* < 0.05) (*n* = 3).

The protease activity of LAB and yeast were assayed using casein as the substrate. Figure 1 showed that LAB and yeast possessed protease activities on the casein substrate ranging from 2.0 to 20 U and 0.1 to 1.0 U, respectively. LN5 was significantly higher than XZ31, which was higher than the others. In most LAB samples, particularly in the XZ31 and LN5 samples, the extracellular protease activities were significantly higher than the intracellular ones (Figure S1). Cell-envelope proteinase (CEP) plays an important role in degrading the casein into oligopeptides during protein utilization by lactic acid bacteria [26]. Many strains of *Lactobacilli* have been proven to possess CEP, which may be one reason for higher intracellular protease activity in the SX1 samples [27]. Despite the limited ability of proteases in yeast strains, there were significant differences between them, with JM4 and JM3 (JM3 > JM4) showing better proteinase activities than the other strains. Different *Saccharomyces cerevisiae* strains (JM4 and JM5) varied greatly in their ability to hydrolyze casein, which indicates that the proteolysis ability of *Saccharomyces cerevisiae* is strain-specific. The protease activity of yeast was also measured using a casein nitrogen-based medium, and no significant proteinase activity was found for the strain (Figure S2). This finding is in agreement with previous results, showing that the yeast strains possess limited ability in secreting external hydrolases [18,28].

**Figure 2:**
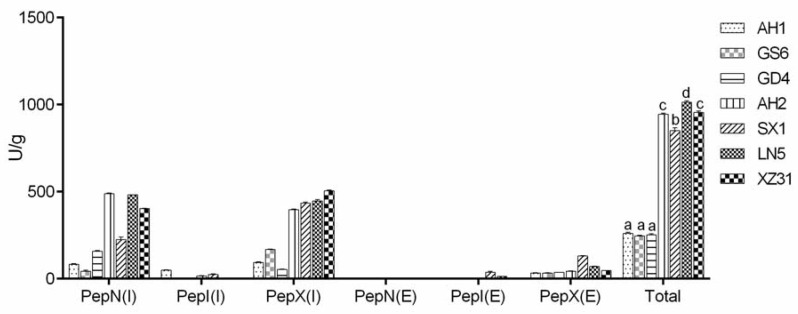
PepN, PepI, and PepX activities of seven lactic acid bacteria strains on Leu-*p*-nitroanilidies, Pro-*p*-nitroanilidies, and Gly-Pro-*p*-nitroanilidies substrates, respectively. (I) and (E) indicate intracellular and extracellular enzyme activities, respectively. Bars that do not share a common lowercase letter differ significantly (*p* < 0.05) (*n* = 3).

Oligopeptide transport systems and peptidases play an important role in protein utilization by lactic acid bacteria [26]. Peptidase activities of lactic acid bacteria were assayed to commercial synthetic substrates. Under our assay conditions, compared with PepI activity, all strains showed higher PepN and PepX activities on Leu-*p*-nitroanilidies and Gly-Pro-*p*-nitroanilidies substrates, respectively. PepN, PepI, and PepX activities were in the range of 40 to 500 U, 1 to 60 U, and 80 to 570 U, respectively (Figure 2). LN5, XZ31, and AH2 (LN5 > XZ31 = AH2) were the strains that showed the highest peptidase activities. In addition, Figure 2 and Figure S3 show a significant difference between the intracellular and extracellular activities, and no PepN activity was found in the extracellular extract.

### 3.2. Proteolysis of Wheat Flour Extracts

**Figure 3:**
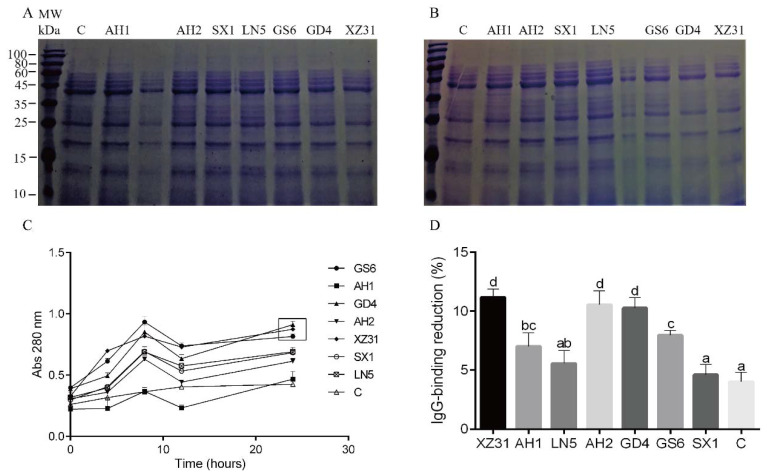
Sodium dodecyl sulfate-polyacrylamide gel electrophoresis (SDS-PAGE) of wheat protein extracts treated with lactic acid bacteria for 0 h (**A**) and 24 h (**B**). (**C**) The same samples in (A, B) were used to quantify the degree of hydrolysis at 280 nm. Each value represents the mean of three independent experiments. (**D**) IgG-binding of wheat protein extracts treated with lactic acid bacteria. Bars that do not share a common lowercase letter differ significantly (*p* < 0.05) (*n* = 3). Control was the mixture without cellular suspension and incubated under the same conditions.

LAB were also screened for the ability to hydrolyze wheat proteins. Through SDS-PAGE analysis, after 24 h of hydrolysis, bands around 15, 25, and 40 KDa were very weak in the GS6, GD4, and XZ31 lanes. There was no significant difference between 0 h and 24 h in control (Figure 3A, B and Figure S4). As shown in the absorbance results (Figure 3C), a reduced pool of short peptides and free amino acids in the supernatant was detected at 8–12 h. These results can be attributed to the limited nutrient supply in the mixture and the lower proteolytic capacity of lactic acid bacteria. During the logarithmic growth, bacteria have a preference for short peptides and free amino acid. After 12 h of hydrolysis, the slow growth of the strain and the accumulation of organic acid, in turn, promoted the accumulation of short peptides and free amino acids and the degradation of high molecular proteins, thereby increasing the content of short peptides and free amino acids [29]. Consistent with the SDS-PAGE analysis, the hydrolysis degree of GD4, XZ31, and GS6 was much higher than in the other samples. A higher substrate specificity for vegetable proteins than for caseins has been reported in some lactic acid bacteria [29], which may be the reason for the different results of the GD4 and GS6 samples for casein and wheat protein hydrolytic activity.

### 3.3. Immunoassay

To compare the capabilities of the selected seven strains for reducing allergenicity, IgG-containing plasma from four sensitized rabbits was used to test the IgG binding capabilities of the hydrolyzed protein extracts. Higher IgG-binding reduction indicates lower allergenicity, and vice versa. Figure 3D shows that five of the identified lactic acid bacteria strains had the ability to reduce protein extract allergenicity. Among them, XZ31, AH2, and GD4 (XZ31 = AH2 = GD4) were the most effective strains, followed by GS6 and AH1. The remarkable hydrolysis of proteins in GD4 and XZ31 samples may be one reason for their reduced IgG-immunoreactivity. The protein fractions were denatured by loading buffer for SDS-PAGE, while the proteins used for allergenic potential analysis (ELISA) were not denatured. This may be a reason for the limited wheat proteolytic and higher IgG-binding reduction capacities of AH2. In addition, the IgG-binding ability is not only related to antigen content, but also to the choice of serum. We also detected the IgG-binding ability of the hydrolysate to human serum and the results showed the highest IgG-binding reduction in samples prepared with the XZ31 strain (data not shown).

### 3.4. Leavening Capacity of Five Yeast Strains

**Figure 4:**
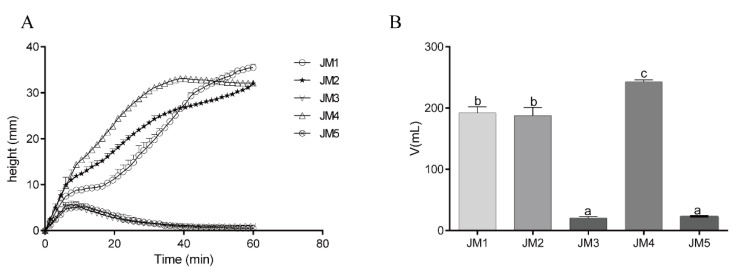
(**A**) Gas release curve. (**B**) Total CO_2_ production of dough. The inocula used for dough fermentation were abbreviated as follows: JM1, *Torulaspora delbrueckiiJM1.* JM2, *Pichia anomalaJM2,* JM3, *Issatchenkia orientalisJM3.* JM4, *Saccharomyces cerevisiaeJM4.* JM5, *Saccharomyces cerevisiaeJM5.* Bars that do not share a common lowercase letter differ significantly (*p* < 0.05) (*n* = 3).

The leavening power of the different yeasts was evaluated by determination of CO_2_ production in dough. Figure 4B shows that CO_2_ production in dough prepared with different yeasts decreased to the order JM4 > JM1 = JM2 > JM5 = JM3. There were also significant differences in leavening capacity between the *Saccharomyces cerevisiae* strains (JM4 and JM5). As shown in the gas release curve (Figure 4A), the CO_2_ production in doughs fermented with JM3 and 5 started quickly, but suddenly dropped before 20 min, indicating a relatively low proofing capacity. There was no significant difference in the content of CO_2_ production between doughs prepared with JM1 and 2 during the test. In addition, fermentation with JM1 and 2 did not show the maximum dough development height under stress during the 1 h test, indicating that JM1 and JM2 have a high fermentation capacity.

## 4. Discussion

Fermentation run by sourdough or isolated fungi and bacteria may markedly reduce wheat allergens and improve the quality of gluten free foods [8,30,31]. We studied Chinese traditional sourdoughs collected from different regions and found that they had different effects on the changes in dough allergenicity. In order to further explore the relationship between sourdough fermentation and allergenicity, seven LAB and five yeast strains were isolated from sourdough and identified by 16S rRNA and 26S rRNA sequencing, respectively. We selected these species, which were commonly identified in sourdough to investigate their potential as hypoallergenic food starters.

Proteolytic systems of LAB and yeasts have been addressed in several studies. The protease activity is mainly associated with the specificity of the strain and enzyme substrate [18,32,33,34,35]. We analyzed the extracellular and intracellular proteolytic activity of LAB and yeast using casein as the substrate. All analyzed LAB were shown to have higher proteolytic activity than yeast strains. Other researchers have also demonstrated limited proteolysis of yeasts [33,36]. Therefore, these strains were not subjected to further protease or peptidase analysis. Many researchers have purified proteinase from *Lactococcus* and *Lactobacillus* strains, which mainly located in the cell envelope [22,37]. The higher extracellular protease activity may due to cell lysis and death, promoting protease release. As indicated by the data in the literature, bacterial proteases possess substrate-specificity characteristics [33]. In this study, the hydrolytic activity of the protease was also investigated against wheat proteins. Strain LN5 showed higher peptide and casein hydrolysis activity, while its hydrolyzing ability to degrade wheat protein was limited. Although the results were not exactly the same, strain XZ31 showed higher proteolytic activity on both casein and wheat protein substrates. In addition, the hydrolysate treated with strain XZ31 exhibited a higher IgG-binding reduction effect on rabbit anti-serum or human anti-serum.

As widely recognized, microbial peptidase activities play an important role in protein utilization during sourdough fermentation [38,39]. Key toxic peptides in gluten have a large proportion of proline, which makes them extremely resistant to pepsin digestion [40], considering this, specific peptidases are necessary for hydrolyzing proline-rich polypeptides. PepI and PepX present in LAB isolated from sourdough were identified as key enzymes to degrade the cyclic structure of proline [8,9,41]. PepN has thoroughly been reported to be a broad specificity aminopeptidase acting on peptides of different amino acid composition and length [39]. The conducted study showed that there was markedly varied peptidase activities among strains. The major part of the strains showed PepI, PepN, and PepX activities, ranging from 2 to 60 U, 40 to 500 U, and 400 to 600 U, respectively (Figure 2). Strains GS6, GD4, and XZ31 showed no PepI activities, and strain XZ31 showed the highest intracellular PepX activity. Consistent with previous studies, no unique strain of lactic acid bacteria possessed the whole pattern of peptidases [22]. These results indicate that further hydrolysis of the immunogenic polypeptides requires the cooperation of different species of LAB. However, it is difficult to completely degrade allergens, and studies have been conducted using wheat and nontoxic flour blends in combination with LAB fermentation to produce reduced allergenicity products [14].

The CO_2_ production of yeasts was closely related to the bread hardness and specific volume with few exceptions. However, a few studies have focused on the leavening capacity of different sourdough yeasts, especially in non-*Saccharomyces* yeasts. In addition, yeasts accumulate thiol, which is related with gluten cross-linking during fermentation. Further research is needed to investigate the relationships between CO_2_ production and thiol levels [42,43,44]. We compared the fermentation ability of five sourdough yeast strains. Under our assay conditions, the tested *Saccharomyces cerevisiae* showed significant difference in CO_2_ production. Strains JM1 and 2 showed a higher leavening capacity than JM5. These results were not consistent with previous studies where *Saccharomyces cerevisiae* exhibited higher leavening abilities than non-*Saccharomyces* yeasts [44,45]. These differences indicate that the leavening ability of yeast is strain-specific and affected by the substrate.

## 5. Conclusions

Previous studies have shown that sourdough lactic acid bacteria as a source of proteolytic enzymes can markedly reduce wheat allergens during dough fermentation. However, the microorganisms in sourdoughs are diverse and related to grain, environment, and fermentation conditions. At present, there are no mixed or single cultures for producing reduced allergenicity wheat products. In this study, we screened 12 strains of lactic acid bacteria and yeasts isolated from Chinese traditional sourdough based on their ability to hydrolyze proteins and ferment dough. Out of these, *Pediococccus acidilacticiXZ31* (XZ31), *Torulaspora delbrueckiiJM1* (JM1), *Saccharomyces cerevisiaeJM4* (JM4) showed superiority over the other strains. Results achieved in the study demonstrate that no unique strain of lactic acid bacteria can cause the complete degradation of allergens. The interaction between different strains may also affect protein hydrolysis and dough fermentation, which is not yet understood, and therefore, further studies are required. Additionally, allergenic potential has to be tested in vivo to support that the selected lactic acid bacteria and yeasts can be considered as unique starter cultures to prepare hypoallergenic wheat products.

## Supplementary Materials

The following are available online at https://www.mdpi.com/2304-8158/9/6/751/s1, Figure S1: Significance test of protease activity value: * p < 0.05. EPT, extracellular proteinases. IPT, intracellular proteinases, Figure S2: Determination of yeast protease activity. JM1, *Torulaspora delbrueckii JM1*. JM2, *Pichia anomala JM2*, JM3, *Issatchenkia orientalis JM3*. JM4, *Saccharomyces cerevisiae JM4*. JM5, *Saccharomyces cerevisiaeJM5*, Figure S3: Significance test of peptidase activity value (including PepN, PepI and PepX): * p < 0.05. Pep(I), intracellular peptidase. Pep(E), extracellular peptidase, Figure S4: Gray value analysis by ImageJ Software (A)bands around 15 kDa (B) bands around 25 kDa (C) bands around 40 kDa (D) the total gray value analysis of main bands. Significant differences are determined with * p < 0.05.
