# Supplementary material for: Screening of Lactic Acid Bacteria and Yeasts from Sourdough as Starter Cultures for Reduced Allergenicity Wheat Products
Source: Foods. 2020 Jun 5;9(6):751. doi: 10.3390/foods9060751 (PMC7353608; doi:10.3390/foods9060751)
Supplement: Supplementary file 1 [file foods-09-00751-s001.pdf]

Screening of lactic acid bacteria and yeasts from sourdough as starter cultures for Reduced Allergenicity wheat products

Wenhui Fu[1,2], Wentong Xue [1,2,*], Chenglong Liu [1,2], Yang Tian [1,2] , Ke Zhang [1,2] and Zibo Zhu[3]

1. Beijing Advanced Innovation Center for Food Nutrition and Human Health, College of Food Science and Nutritional Engineering, China Agricultural University, Beijing 100083

2. College of Food Science and Nutritional Engineering, China Agricultural University, Beijing 100083

3. College of Civil engineering and architecture, Henan University of Technology, Zhengzhou 450000

Supplementary Material

1. Supplementary Method

Rabbit antisera

Healthy 4.5-5 month old male New Zealand white rabbit (2.5 ± 0.2 kg) were obtained from Vital River (Beijing, China). Animals were maintained under standard laboratory conditions in a pathogen-free environment and on a wheat-free diet.

In brief, blood samples were collected from the auricular vein (n=4) for control before immunization. Four rabbits were subcutaneously immunized (2 mL / head) with an equal amount of wheat proteins mixed with complete Freund’s adjuvant (sigma) and boosted one times at 2-week intervals with the same amount of proteins plus complete Freund’s adjuvant. A booster dose was injected 4 weeks later using the same amount of protein with Freund’s incomplete adjuvant. The antibody titer of immunized rabbit was detected using indirect ELISA on day 10 after immunization. Blood samples were obtained from the heart and polled, aliquoted, and stored at -20 °C until use.

2. Supplementary Figures

Fig. S1. Wenhui Fu

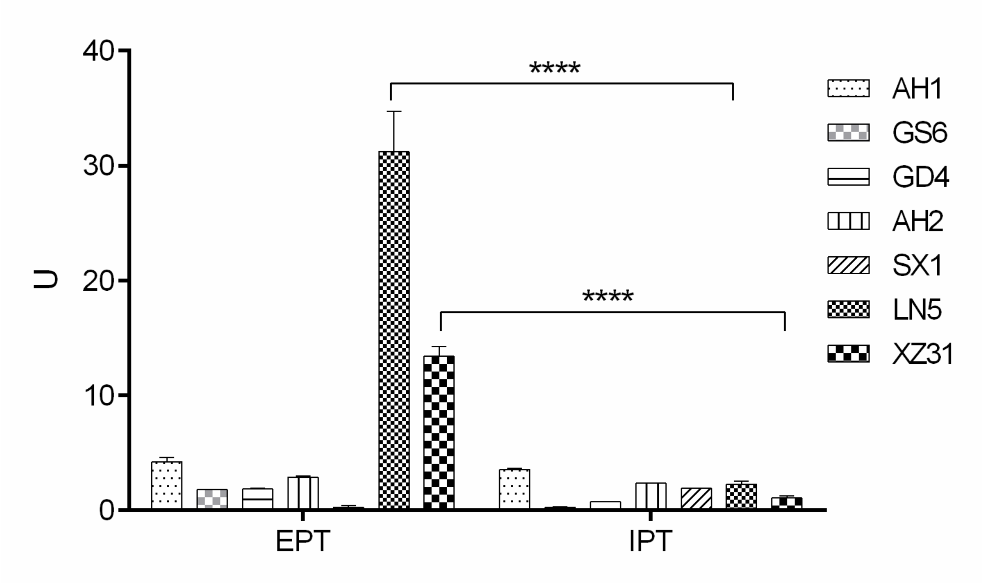

Significance test of protease activity value: *$p < 0.05$. EPT, extracellular proteinases. IPT, intracellular proteinases.

Fig. S2. Wenhui Fu

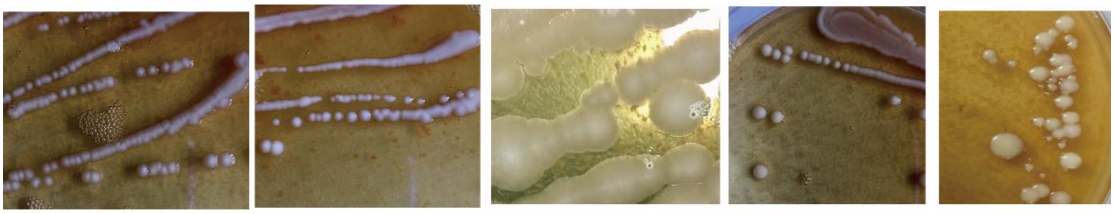

JM1 JM2 JM3 JM4 JM5

Determination of yeast protease activity. JM1, *Torulaspora delbrueckiiJM1*. JM2, *Pichia anomalaJM2*, JM3, *Issatchenkia orientalisJM3*. JM4, *Saccharomyces cerevisiaeJM4*. JM5, *Saccharomyces cerevisiaeJM5*.

Fig. S3. Wenhui Fu

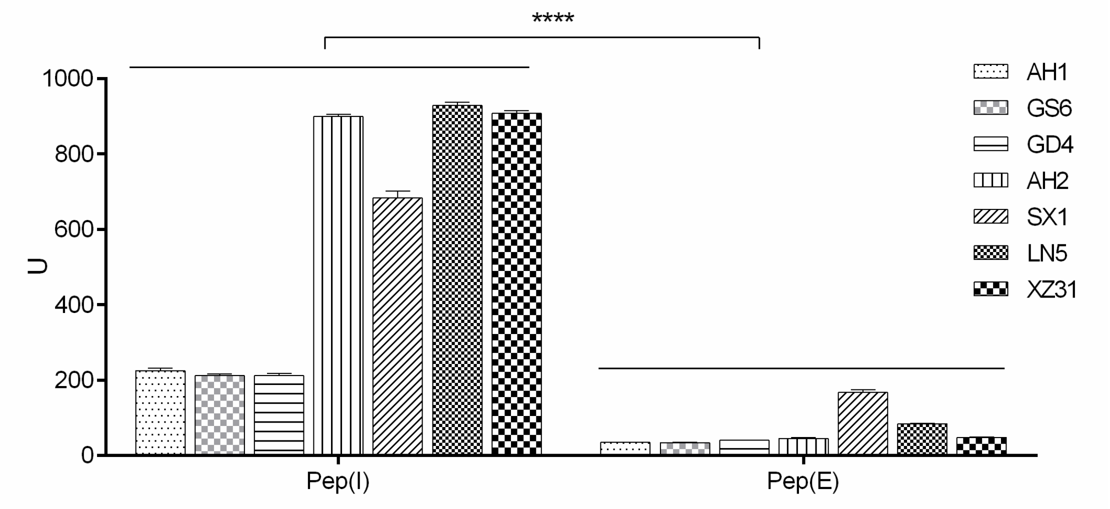

Significance test of peptidase activity value (including PepN, PepI and PepX): *p < 0.05. Pep(I), intracellular peptidase. Pep(E), extracellular peptidase.

Fig. S4. Wenhui Fu

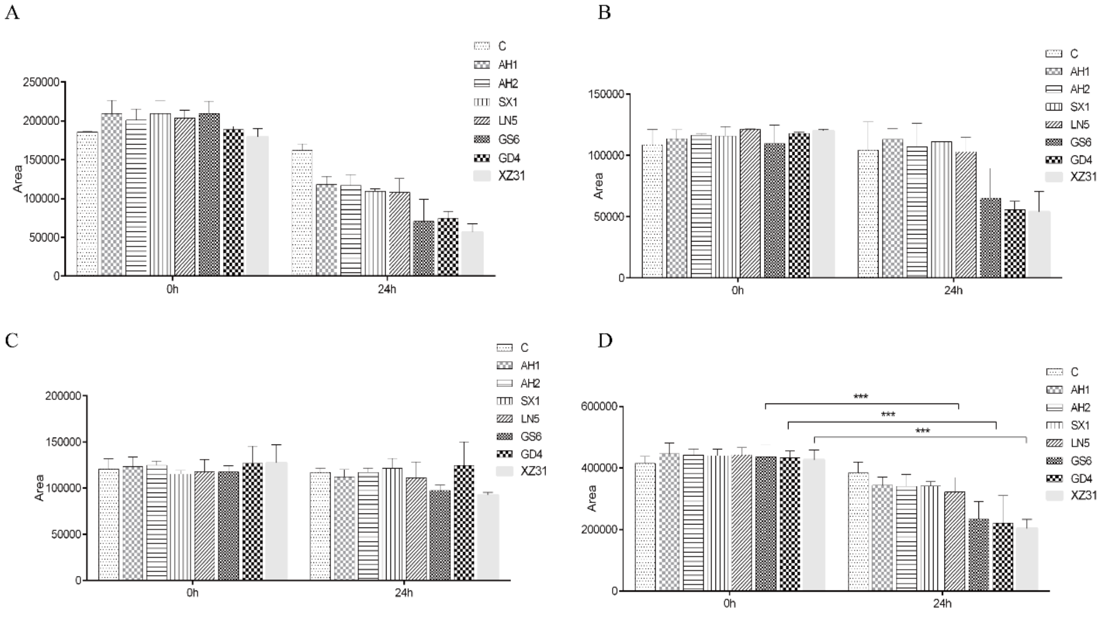

Gray value analysis by ImageJ Software.（A）bands around 15 kDa (B) bands around 25 kDa (C) bands around 40 kDa (D) the total gray value analysis of main bands. Significant differences are determined with *p < 0.05.
